# Retraction: 17β-Estradiol protects neural stem/progenitor cells against ketamine-induced injury through estrogen receptor β pathway

**DOI:** 10.3389/fnins.2026.2017403

**Published:** 2026-10-12

**Authors:** Frontiers Editorial Office

The journal retracts the 30 September 2020 article cited above.

Following publication, concerns were raised regarding the integrity of the images in the published figures. The authors failed to provide a satisfactory explanation during the investigation, which was conducted in accordance with Frontiers' policies.

This retraction was approved by the Chief Editors of Frontiers in Neuroscience and the Chief Executive Editor of Frontiers. The authors received a communication regarding the retraction and had a chance to respond. This communication has been recorded by the publisher.

